# Nutrient and Metabolic Sensing in T Cell Responses

**DOI:** 10.3389/fimmu.2017.00247

**Published:** 2017-03-09

**Authors:** Jun Wei, Jana Raynor, Thanh-Long M. Nguyen, Hongbo Chi

**Affiliations:** ^1^Department of Immunology, St. Jude Children’s Research Hospital, Memphis, TN, USA

**Keywords:** glucose, amino acid, lipid, oxygen, energy, T cell response

## Abstract

T cells play pivotal roles in shaping host immune responses in infectious diseases, autoimmunity, and cancer. The activation of T cells requires immune and growth factor-derived signals. However, alterations in nutrients and metabolic signals tune T cell responses by impinging upon T cell fates and immune functions. In this review, we summarize how key nutrients, including glucose, amino acids, and lipids, and their sensors and transporters shape T cell responses. We also briefly discuss regulation of T cell responses by oxygen and energy sensing mechanisms.

## Introduction

T cells express specific T cell receptors (TCRs) and play pivotal roles in cell-mediated immunity during infection, autoimmune diseases, and cancer. Conventional αβ T cells are divided into two major lineages based on the exclusive expression of the co-receptor CD8 or CD4 (1). Upon cognate antigen stimulation, naïve CD8^+^ T cells expand and differentiate into cytotoxic effector cells to clear infected or malignant cells. After this rapid expansion, effector CD8^+^ T cells undergo contraction, and only a small subset of them eventually forms the memory population (2). Likewise, naïve CD4^+^ T cells are activated by cognate antigens and undergo proliferation and functional specialization. In response to an instructive cytokine milieu, naïve CD4^+^ T cells differentiate into functionally distinct T helper cells, including T_H_1, T_H_2, T_H_17, and T_FH_ effector cells, and immunosuppressive T_reg_ cells (3). Although immune receptors, signaling proteins and transcriptional factors allow T cells to sense and transduce antigenic and inflammatory signals, emerging studies highlight that cellular metabolism is dynamically regulated to control T cell survival, proliferation, and function (4). Nutrients are the basis of cellular metabolism, with glucose, amino acids, and lipids playing crucial roles in immune signaling and immune cell functions (5). Because T cell priming and function occur within discrete microenvironments, they must adapt to changes in nutrient and environmental signals to mount effective adaptive immune responses.

Nutrient-sensing systems, composed of sensors, transporters, and signaling proteins, are utilized by cells to monitor and respond to fluctuations in environmental nutrient levels. Several evolutionarily conserved nutrient-sensing systems have been identified (6, 7). Recently, extensive efforts have been made to dissect mechanisms of nutrient sensing in T cells. This review article first discusses the regulation and function of glucose metabolism in T cell responses. Second, we summarize recent findings on how specific amino acids and amino acid transporters control T cell responses. Third, we summarize the contributions of fatty acid (FA) and cholesterol sensing to T cell-mediated immunity. Finally, we briefly discuss how T cells adapt to oxygen levels and energy stress to maintain their functions.

## Glucose Sensing

**Figure 1:**
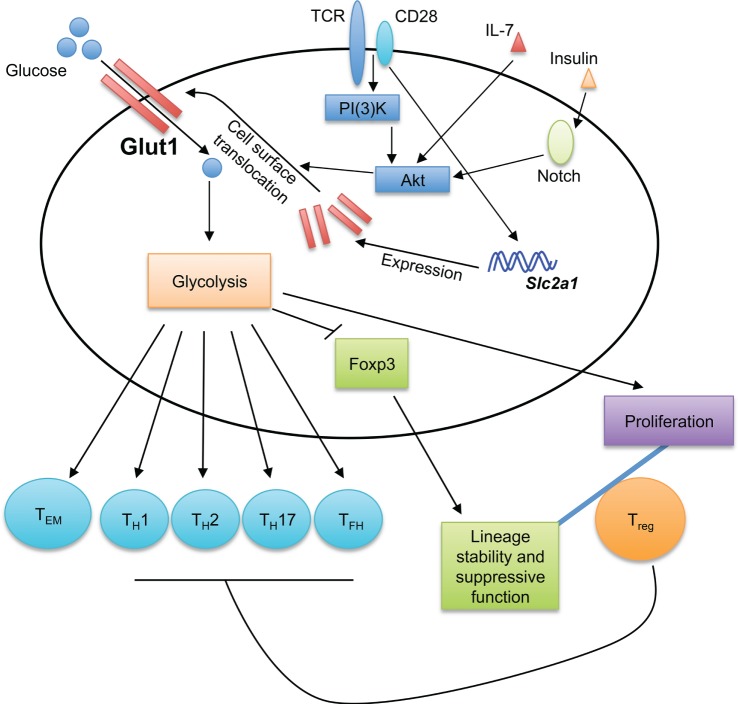
**Glucose sensing shapes T cell immune responses**. Glucose transporter 1 (Glut1) transports glucose into T cells to fuel glycolysis. The generation of effector T cells, including T_H_1, T_H_2, T_H_17, and T_FH_ cells, is dependent on Glut1 expression and glycolytic metabolism. While Glut1 expression and glycolysis promote T_reg_ cell proliferation, they impair T_reg_ cell lineage stability and suppressive function. Thus, Glut1 expression provides a key mechanism to shape T cell responses. Glycolysis also promotes the formation of effector memory CD8^+^ T cells (T_EM_). TCR and CD28 co-stimulation potently induces Glut1 expression, while the translocation of Glut1 from the cytoplasm to T cell surface is induced by various stimuli, including TCR-CD28, IL-7, and insulin.

Glucose is a major cellular nutrient that serves as a critical fuel for cellular adenosine triphosphate (ATP) generation and precursor for biosynthetic pathways. T cell activation, subset differentiation, and effector function are tightly regulated by glucose metabolism as detailed below (Figure 1).

### Glucose Metabolism in Conventional T Cell Activation, Differentiation, and Memory Formation

Activated T cells use glucose metabolism to fulfill the metabolic requirements for rapid proliferation and biosynthesis supporting cellular growth and differentiation (4). Such demands are met, in part, by upregulation of glucose transporter 1 (Glut1) during T cell activation. Naïve T cells express low levels of Glut1 on the cell surface, but Glut1 expression and surface localization are significantly increased in activated T cells (8–10). Several upstream signals regulate Glut1 expression in T cells. TCR and CD28 co-stimulation induces the expression and surface localization of Glut1 in T cells (10). The phosphoinositol-3 kinase (PI(3)K)-Akt pathway triggers the translocation of Glut1 from cytoplasm to T cell surface (9, 10). Other stimuli also increase surface Glut1 levels on T cells; for example, IL-7-induced STAT5 signaling and downstream Akt activation induce Glut1 trafficking to the cell surface in both naïve and activated T cells (11). Increased surface expression of Glut1 is observed on memory CD4^+^ T cells upon insulin stimulation through a mechanism that requires Notch-Akt signaling (12). In human CD4^+^ T cells, the *SLC2a1* (encodes for human Glut1) mRNA transcript is targeted by miR-150, whose expression is downregulated by co-stimulation with the complement receptor CD46 (13). CD46 signaling is also important for effective T_H_1 cell differentiation by potentiating TCR-driven Glut1 expression and surface translocation (14).

Extensive efforts have been made to determine the role of Glut1 in T cell responses. While Glut1 overexpression does not alter T cell development, it enhances activated T cell proliferation and growth (10, 15), and generation of T_FH_ cells (16). Also, T_H_1, T_H_2, and T_H_17 cells express high levels of Glut1 and are highly glycolytic (15, 17). Consistent with this observation, conditional deletion of *Slc2a1* (encodes for mouse Glut1) in the T cell compartment leads to defective generation of T_H_1, T_H_2, and T_H_17 cells both *in vitro* and *in vivo* (8). How Glut1 expression and glucose metabolism specifically contribute to the functional specialization of effector CD4^+^ T cell subsets requires further investigation. Transgenic expression of Glut1 leads to an accumulation of activated/memory phenotype T cells *in vivo*, suggesting a role of glucose metabolism in T cell memory responses (10, 15). Memory T cells have reduced glycolysis as compared to effector T cells (18), but Glut1 expression and glucose uptake are essential for CD4^+^ memory T cell maintenance (12). A recent study demonstrates that constitutive glycolysis promotes the formation of effector memory CD8^+^ T cells (T_EM_) in a murine genetic model with T cell-specific deletion of a key hypoxia-inducible factor 1α (HIF1α) negative regulator, Von Hippel-Lindau (VHL) (19). Consistent with these observations, Glut1 expression favors human T_EM_ formation (20). Moreover, the rapid acquisition of effector functions by human T_EM_ requires immediate-early glycolysis upon activation (21). Thus, Glut1 and glucose metabolism are essential for effector and T_EM_ responses. Further studies are required to reveal the underlying molecular mechanisms.

Recent studies have uncovered important roles of glucose competition between tumor cells and effector T cells within the tumor microenvironment (22–24). Similar to T cells, tumor cells rewire their metabolism to greatly increase glucose uptake and aerobic glycolysis, which is known as the Warburg Effect (25). The functional state of tumor-infiltrating T cells is positively correlated with their glycolytic activity and glucose availability within tumor tissues (23, 24). Due to the limited glucose levels in the tumor microenvironment, tumor-infiltrating T cells produce less IFN-γ than T cells taken from glucose-replete environments (23). Mechanistically, this is linked to the ability of glyceraldehyde-3-phosphate dehydrogenase (GAPDH) to serve as both a glycolytic enzyme and RNA-binding protein (26, 27). When aerobic glycolysis is inhibited, GAPDH binds to the *Ifng* (encodes for IFN-γ) mRNA transcript to suppress its translation (28). Aerobic glycolysis also plays a pivotal role in sustaining TCR-mediated calcium-NFAT signaling to maintain T cell effector functions (24). Specifically, phosphoenolpyruvate (PEP) generated during glycolysis maintains cytosolic calcium levels by suppressing sacro/endoplasmic reticulum calcium ATPase activity (24). Notably, increasing PEP production enhances anti-tumor T cell responses (24). Removing the metabolic restrictions in T cells may also contribute to the therapeutic effects of checkpoint blockade therapies, including anti-CTLA4, anti-PD-1, and anti-PD-L1 antibody administrations, since those treatments restore glucose levels within tumors and glycolytic metabolism in T cells (23). More research is needed to determine the therapeutic potential of targeting the components of glucose sensing and metabolism in T cells in cancer patients.

### Glucose Metabolism in T_reg_ Cells

The roles of glycolytic metabolism have also been investigated in suppressive Foxp3^+^ T_reg_ cells. Murine T_reg_ cells express comparable levels of Glut1 as naïve T cells but lower levels of Glut1 than effector T cells (8, 15). Such regulation of Glut1 expression is partially dependent on elevated AMP-activated protein kinase (AMPK) activation in T_reg_ cells (15). Foxp3, the master transcription factor that governs T_reg_ cell differentiation and function, limits Glut1 expression through inhibiting Akt (29). Glut1 deficiency does not affect T_reg_ cell suppressive function but increases the proportion of T_reg_ cells in the peripheral CD4^+^ T cell compartment (8). In contrast, T_reg_ cells with aberrant increases in glucose metabolism tend to lose their lineage stability. Indeed, murine T_reg_ cells with elevated Glut1 expression have reduced CD25 and Helios expression and are unable to maintain Foxp3 expression and suppressive function in a murine inflammatory bowel disease model, indicative of reduced T_reg_ cell stability (30). These results are consistent with recent findings that aberrant glycolysis is detrimental to T_reg_ cell lineage stability and functional integrity (31–33). Of note, proliferating human and murine T_reg_ cells have elevated glucose uptake and glycolysis than non-dividing T_reg_ cells, and glycolysis contributes to the functional differentiation of human T_reg_ cells by inducing FOXP3 expression (34, 35). These studies highlight a pivotal role of glucose metabolism in balancing the proliferation and suppressive function of T_reg_ cells, which is likely important for controlling effector and suppressive T cell responses during infection and inflammation.

## Amino Acid Sensing

**Figure 2:**
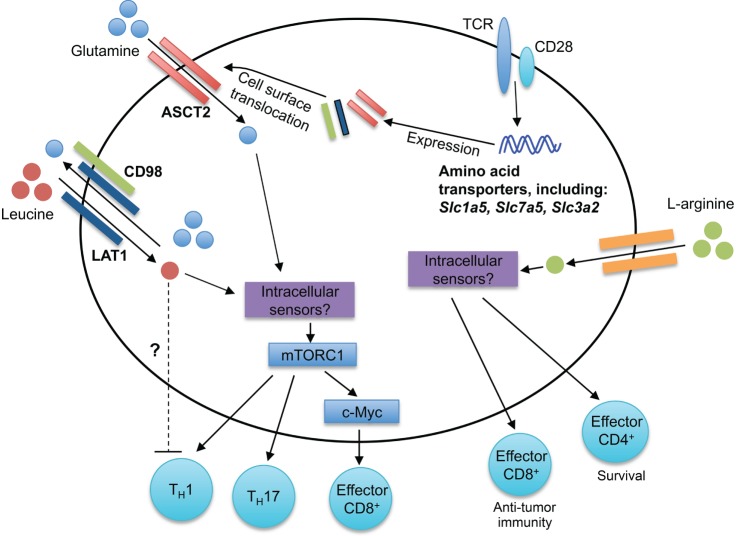
**Amino acid sensing modulates T cell responses**. Antigen-driven activation of T cells through TCRs upregulates expression of many amino acid transporters, including the leucine and glutamine transporters LAT1, ASCT2, and CD98. LAT1 associates with CD98, forming a bidirectional transporter for leucine and glutamine. The intracellular sensors of leucine and glutamine in T cells remain unknown. mTORC1 is activated downstream of intracellular amino acids, leading to the regulation of CD4^+^ T cell differentiation (T_H_1 and T_H_17) and CD8^+^ T cell effector responses. Elevated intracellular l-arginine levels promote effector CD8^+^ T cell anti-tumor immunity and effector CD4^+^ T cell IFN-γ production and survival. Although the intracellular mediators critical for arginine sensing are unclear, three potential sensors are BAZ1B, PSIP1, and TSN.

Amino acids are the building blocks for protein synthesis, and their uptake into cells is critical for cellular function. During cellular division, the influx of amino acids is especially critical to meet the increased demands for protein synthesis. Furthermore, amino acids can serve as sources for metabolites that enter into metabolic processes, such as the tricarboxylic acid (TCA) cycle. Such energy-demanding cellular processes must be tightly regulated, requiring the sensing of extracellular and intracellular amino acid abundance. Recent studies have begun to identify specific amino acids and amino acid transporters that are critical in regulating T cell homeostasis and function (Figure 2).

### Leucine and Glutamine

Amino acids are critical for efficient T cell activation and proliferative responses (36). The SLC7 amino acid transporter family contributes to the uptake of amino acids by T cells. The SLC7 amino acid transporter family is comprised of the cationic amino acid transporters (SLC7A1-4, CAT1-4, and SLC7A14) and the glycoprotein-associated amino acid transporters (SLC7A5-13, SLC7A15) (37). Antigen signaling through the TCR increases phenylalanine, leucine, and glutamine uptake into CD8^+^ T cells (38, 39). Furthermore, the expression of many SLC7 transporters, including *Slc7a5* (encodes for LAT1), is upregulated during activation in both mouse and human T cells (38, 40), which may be mediated by TCR-induced ERK/MAPK signaling (39). LAT1 associates with CD98 (encoded by *Slc3a2*) to form a high-affinity transporter that facilitates the bidirectional influx of leucine and efflux of glutamine from cells (38, 41). LAT1 is not required for normal T cell development; however, LAT1*-*deficient CD8^+^ T cells have impaired primary effector expansion following antigenic stimulation (38). Mechanistically, leucine uptake induces activation of mechanistic target of rapamycin complex 1 (mTORC1) in effector CD8^+^ T cells and promotes c-Myc expression (38). Furthermore, the loss in c-Myc expression in CD8^+^ T cells correlates with impaired glucose, glutamine, and arginine uptake and impaired glycolytic metabolism, which promote proper CD8^+^ T cell effector function (38, 42). Thus, leucine is a positive regulator of peripheral CD8^+^ T cell functionality. There is much left to be understood how amino acid sensing regulates CD8^+^ T cell responses. For instance, while glutamine receptor ASCT2 (encoded by *Slc1a5*) is dispensable for CD8^+^ T cell development (41), how ASCT2 regulates CD8^+^ T cell effector and memory responses has not been explored.

Both leucine and glutamine are positive regulators of CD4^+^ T cell differentiation into T_H_1 and T_H_17 cells. Indeed, the absence of LAT1 expression impairs T_H_1 and T_H_17 cell differentiation *in vitro* (38), and elevated glutamine levels favor T_H_1 and T_H_17 cell differentiation *in vitro* (41). Consistent with these observations, ASCT2 is important for T_H_1 and T_H_17 cell differentiation (41). Likely due to the bidirectional transport of glutamine and leucine *via* LAT1, activated ASCT2-deficient CD4^+^ T cells also have decreased leucine uptake (41, 43). Interestingly, highly elevated leucine levels favor T_H_17 differentiation, but impair T_H_1 differentiation *in vitro*, indicating that high concentrations of leucine may negatively regulate T_H_1 differentiation (41). Glutaminolysis plays crucial roles in replenishing the TCA cycle under conditions of aerobic glycolysis and supports T cell activation (42). Furthermore, the glutamine-derived metabolite alpha-ketoglutarate promotes T_H_1 differentiation, potentially through enhancing mTORC1 signaling (44). Unlike T_H_1 and T_H_17 cells, T_H_2 and T_reg_ cells do not require the expression of LAT1 or ASCT2 for their differentiation *in vitro* (38, 41), likely due to their altered dependency on mTOR signaling (45). Indeed, T_reg_ cell differentiation is favored during glutamine deprivation (44). Thus, leucine and glutamine likely act in concert to regulate the threshold of mTORC1 signaling and subsequent CD4^+^ T cell differentiation.

### Arginine

Arginine is transported into cells through the cationic transporters CAT1-4 (46), where it is metabolized by arginase 1 or nitric-oxide synthase 2 (46). Myeloid-derived suppressor cells, which are elevated in tumor microenvironment and other disease states, consume and deplete extracellular arginine (46). These arginine-depleted environments impair T cell proliferation (47–50). Arginine is also directly metabolized in T cells (51). Activated T cells have increased l-arginine metabolism mediated by arginase 2, which contributes to enhanced CD4^+^ and CD8^+^ T cell survival and anti-tumor immunity (51). Interestingly, *Arg2*-deficient CD4^+^ and CD8^+^ T cells have elevated levels of intracellular l-arginine, which correlates with increased survival after IL-2 withdrawal (51), suggesting that additional arginine-sensing pathways contribute to T cell survival. While the mechanisms of how arginine is sensed within T cells remain elusive, BAZ1B, PSIP1, and Translin are potential arginine sensors that promote T cell survival (51). Interestingly, these survival effects appear to be independent of modulating mTORC1 activation, which is a known downstream sensor of arginine in other cell lineages (52–54). Thus, regulation of arginine availability and sensing is critical for T cell functionality and combating environmental insults, such as infections and tumors. Future studies can explore how lysine and ornithine, whose uptake is also CAT dependent, contribute to T cell responses.

### Serine

Serine is a non-essential amino acid that is synthesized intracellularly from the glycolytic intermediate 3-phosphoglycerate, or directly transported into cells (55). Recent work reveals that serine metabolism is enhanced in activated T cells and provides intracellular glycine and one-carbon units for purine nucleotide biosynthesis, thereby supporting T cell proliferation (56). Serine uptake is critical for effector T cell responses, as serine and glycine-limiting conditions impair anti-CD3/CD28 antibody-driven CD4^+^ and CD8^+^ T cell proliferation *in vitro* (56). Furthermore, mice on a serine and glycine-restricted diet have impaired antigen-driven CD8^+^ effector T cell expansion and pathogen clearance (56). These findings elucidate a novel role for serine metabolism in effector T cell responses.

### Tryptophan

Indoleamine 2,3-dioxygenase (IDO) catabolizes the amino acid tryptophan, resulting in localized tryptophan depletion. Tryptophan depletion is then sensed by general control non-derepressible 2 (GCN2), which has a high affinity for uncharged tRNAs that accumulate when intracellular amino acid abundance is low. GCN2 binding to uncharged tRNAs induces GCN2 kinase activity, which subsequently inhibits protein translation *via* the phosphorylation of eIF2α (6). It has been demonstrated that GCN2 sensing of amino acid deprivation in activated T cells promotes T cell survival, limits T cell proliferation, and induces T cell anergy (36, 57). In contrast to these findings, a recent study has shown that both control and GCN2-deficient CD4^+^ and CD8^+^ T cells have impaired proliferation when tryptophan, arginine, lysine, leucine, or asparagine levels are low, suggesting additional pathways contribute to sensing amino acid deprivation in T cells (58). IDO may also influence T cell responses independently of GCN2 kinase activity by altering the inflammatory environment. Indeed, tryptophan metabolites promote T_reg_ cell differentiation indirectly by increasing TGF-β production by dendritic cells (DCs) (59). Furthermore, blocking IDO promotes IL-6 production by plasmacytoid DCs, which enables skewing of CD4^+^ T cells toward the T_H_17 cell subset and suppression of T_reg_ cell development (60, 61). Consequently, IDO deficiency promotes T_H_17 differentiation and exacerbates experimental autoimmune encephalomyelitis (EAE), a murine model of human multiple sclerosis (59). Thus, the IDO-tryptophan degradation pathway plays a regulatory role in local inflammation driving T_H_17 cell responses.

## Lipid Sensing

**Figure 3:**
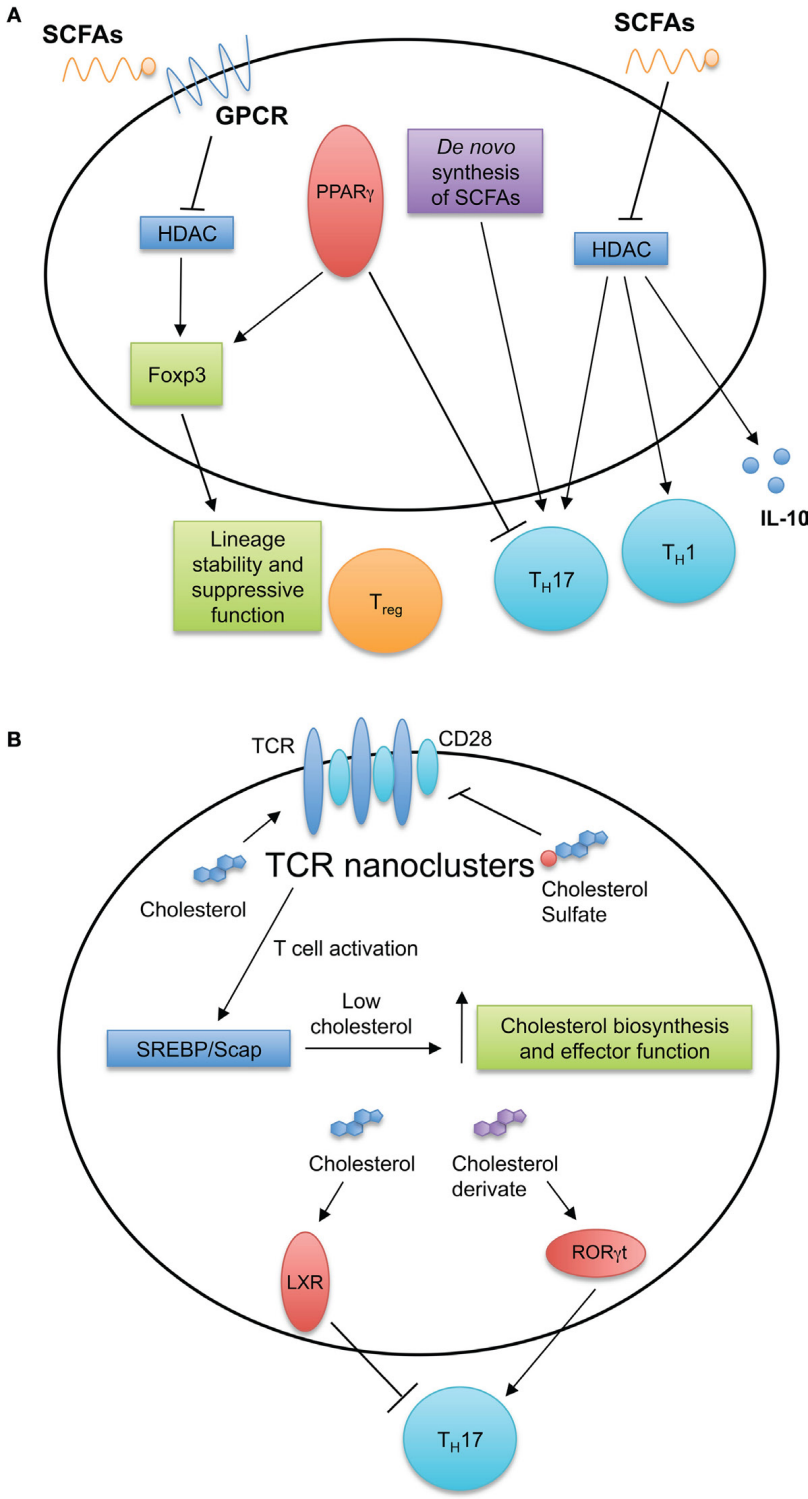
**Fatty acid (FA) and cholesterol sensing in T cell immune responses**. **(A)** FA metabolism and sensing serve multiple roles in T cell biology. Short-chain fatty acids (SCFAs) diffuse passively or are transported into cells by G-protein-coupled receptors. SCFAs can inhibit histone deacetylases and induce T_H_1 and T_H_17 cell differentiation and IL-10 production. *De novo* FA synthesis is required for T_H_17 generation. Intracellular FAs are recognized by peroxisome proliferator-activated receptors (PPARs). Expression of PPARγ is important for visceral adipose tissue T_reg_ cell accumulation and function, while antagonizing PPARγ by chemical drug promotes T_H_17 development. **(B)** Cholesterol and cholesterol sulfates regulate T cell receptor (TCR) nanoclustering and signaling within the T cell. After stimulation of TCRs, T cells upregulate the cholesterol biosynthesis pathway through the SREBP/SCAP axis. This increase in cholesterol biosynthesis is important for acquiring T cell effector function. Additionally, intracellular sterols serve as ligands for intracellular receptors and transcription factors, such as liver X receptor (LXR) and RORγt to modulate T_H_17 development.

Lipid molecules, including FAs and sterols, are essential for cellular function, serving important functions in membrane biosynthesis and intracellular signaling. Several studies indicate roles for cholesterol and FAs in activation, differentiation, and function of T cells as discussed below (Figure 3).

### Fatty Acids

The basic structure of a FA is a carboxylic acid linked to a saturated or unsaturated aliphatic chain. FAs are categorized broadly into groups based on the length of the aliphatic chain. Short-chain fatty acids (SCFAs) have 2–6 carbons; medium chain fatty acids have 7–12 carbons; and long chain fatty acids (LCFAs) have more than 12 carbons (62). Some FAs are generated within the cell through *de novo* synthesis; however, many important FAs cannot be synthesized and are obtained only through dietary sources. For instance, when dietary fats and fibers are catabolized in the liver and intestines, respectively, free fatty acids (FFAs) are generated and circulate through the blood (62). Although certain FFAs can diffuse across the plasma membrane into the cytosol, most require transport facilitated by surface receptors. LCFAs and oxidized FAs are transported across the membrane by fatty acid translocase (FAT or CD36) (63). Additionally, several G protein receptors bind FAs of various lengths—GPCR40 (free fatty acid receptor 1) and GPCR120 bind to LCFAs; GPCR84 recognizes MCFAs; GPCR43 (FFA2) and GPCR41 (FFA3) bind to SFCAs (62).

Several studies have revealed roles for SFCAs and their transporters in T cell responses. The breakdown of dietary fibers into acetate, butyrate, and propionate by intestinal bacteria plays an important role in controlling T cell inflammatory responses. For instance, within the intestines, these SCFAs bind the GPCR43 receptor on T_reg_ cells and promote their differentiation and function to maintain intestinal homeostasis (64, 65). Mechanistically, butyrate inhibits histone deacetylase (HDAC) activity, subsequently enhancing histone acetylation in the *Foxp3* promoter to promote stable Foxp3 expression (64, 66, 67). Interestingly, these intestine-derived SCFAs also promote the generation of T_reg_ cells in distal tissues (66) and shape inflammatory responses within the central nervous system, kidneys, and lungs (68–71). For example, during an acute *Listeria monocytogenes* infection, serum levels of acetate increase, which is required for optimal memory CD8^+^ T cell formation (72). The increase in intracellular acetate enhances glycolytic activity necessary for a rapid recall response by CD8^+^ T cells (72). Mechanistically, the accumulation of acetate drives GAPDH acetylation to increase glycolytic flux (72). Furthermore, SCFA-mediated inhibition of HDAC activity increases the acetylation of S6K. This process promotes T_H_1 and T_H_17 cell differentiation and induces IL-10 production by CD4^+^ T cells (70). These studies show the pleiotropic functions of SCFAs in immune responses and provide evidence for targeting FA sensing pathways to modulate T cell responses.

Intracellular FAs are also recognized by peroxisome proliferator-activated receptors (PPARs). The PPAR family of nuclear receptors has three isoforms: PPARα, PPARδ, and PPARγ. After recognizing their ligands, PPARs associate with the retinoid X receptor heterodimer, which binds PPAR-responsive elements to promote gene transcription (73). PPARγ is an important regulator of effector T cell functions, as PPARγ-deficient CD4^+^ T cells cannot proliferate and survive in a lymphopenic environment and hence are less capable of inducing autoimmunity and graft-versus-host disease in mice (74). Antagonizing PPARγ promotes T_H_17 cell differentiation and increases the severity of EAE (75, 76). Excessive T_H_17 cell differentiation might also be linked to the role of PPARγ in T_reg_ cell differentiation. Indeed, PPARγ is essential for the maintenance and accumulation of T_reg_ cells in adipose tissue (77). Thus, PPARγ serves as a key regulator of CD4^+^ T cell function and differentiation.

### Cholesterol

To facilitate ligand recognition and initiate efficient signaling, several TCRs must oligomerize into nanoclusters (78). This process is controlled by cholesterol and its derivatives. Cholesterol-facilitated nanoclustering of TCRs enhances the avidity of the TCR toward its ligand (79). Conversely, cholesterol sulfate and cholesterol esters antagonize TCR function by displacing cholesterol and disrupting the formation of nanoclusters (80). Interestingly, cholesterol may serve temporal or spatial roles in TCR signaling. Cholesterol is an allosteric inhibitor of the TCRβ transmembrane domain and hence limits spontaneous TCR activation; however, the spontaneous unbinding of cholesterol in this region switches the conformation from inactive to active, allowing for the transmission of TCR signals (81). Therefore, cholesterol level in the plasma membrane of T cells is important for regulating TCR activity, which may contribute to T cell development and functions under different conditions.

Intracellular concentrations of cholesterol play key roles in both development and effector functions of T cells. Inhibition of the esterification enzyme acetyl-coenzyme A acetyltransferases 1 increases cholesterol content in the plasma membrane of CD8^+^ T cells, which augments TCR nanoclustering and subsequently increases effector CD8^+^ T cell functions (82). Furthermore, deletion of ATP-binding cassette sub-family G member 1 (ABCG1), a protein promoting cholesterol efflux from cell, leads to an accumulation of intracellular cholesterol in T_reg_ cells and increase in T_reg_ cell frequency. Mechanistically, loss of ABCG1 and subsequent disruption in intracellular cholesterol distribution decreases mTOR activity and increases STAT5 signaling, thereby promoting T_reg_ cell formation (83). These data demonstrate that intracellular cholesterol levels are essential for T cell function.

Cholesterol and its derivatives can also serve as intracellular ligands to influence T cell differentiation and function. Specifically, cholesterol derivatives control T_H_17 cell differentiation by modulating liver X receptor (LXR) and retinoic acid responsive-related orphan receptor γt (RORγt) activity. LXR engagement antagonizes T_H_17 cell development by mediating the binding of sterol response element-binding protein (SREBP)1 to the *Il17a* locus and blocking the upregulation of aryl hydrocarbon receptor (AHR) necessary to induce *Il17a* transcription (84). Additionally, many cholesterol biosynthetic intermediates and derivatives are natural ligands for RORγt, the master transcription factor for T_H_17 cell development (85–87). These derivatives include oxysterol, desmosterol, and many cholesterol intermediates generated between lanosterol and zymosterol biosynthesis. Pharmaceutical or genetic disruption of these metabolites limits T_H_17 cell generation and ameliorates the severity of EAE (85–87). If and how cholesterol intermediates influence other effector CD4^+^ T cell differentiation require further investigation.

### FA and Cholesterol Biosynthesis

The ability to sense cholesterol and generate *de novo* cholesterol is also critical for the activation and effector functions of T cells. Cholesterol synthesis is regulated within the cell by the transcription factor, SREBP2, and its chaperone SREBP cleavage activating protein (SCAP), which helps sense intracellular cholesterol levels (88). T cell activation induces the activation of SULT2B1, an oxysterol-metabolizing enzyme, which indirectly enhances SREBP2-dependent cholesterol synthesis by limiting oxysterols that activate LXR. Thus, SULT2B1 limits cholesterol export from the cell by blocking the LXR-dependent upregulation of cholesterol-modifying enzymes and transporters (89). Intrinsic SCAP expression is required for T cell proliferation and effector function (90). While SCAP-deficient T cells develop normally and are capable of undergoing homeostatic proliferation, they do not generate the requisite lipids for cell growth induced by TCR stimulation (90). Whether SCAP deficiency affects TCR nanonclustering is unknown, but early TCR signaling is not impaired in the absence of SCAP despite these cells having reduced total cholesterol.

TCR stimulation-induced lipid biosynthesis is dependent on mTORC1 activation (90). Naïve CD4^+^ T cells deficient in Raptor, an obligatory component of mTORC1, neither efficiently upregulate the expression of SREBP or other cholesterol-related genes nor appropriately grow and proliferate upon TCR and CD28 co-stimulation. These defects in cholesterol synthesis, along with compromised glycolysis and oxidative phosphorylation, are correlated with a reduced capacity to differentiate into effector CD4^+^ T cells (91). Raptor-deficient T_reg_ cells also have reduced cholesterol-related gene expression, which severely compromises T_reg_ cell proliferation and suppressive function (92).

Of interest, the energy production and suppressive capacity of T_reg_ cells are also linked to FA oxidation (FAO) (15), similar as memory CD8^+^ T cells (18). Memory CD8^+^ T cells fuel FAO via *de novo* lipid synthesis to support their homeostasis (18, 93). Additionally, *de novo* FA synthesis is crucial for the regulation of T_reg_ and T_H_17 cell differentiation. Inhibition of acetyl-CoA carboxylase-1 (ACC1), a key enzyme for FA synthesis, promotes T_reg_ cell and inhibits T_H_17 cell differentiation. Mechanistically, ACC1 deficiency limits FA synthesis and glycolysis needed for T_H_17 cell development (94). While the low level of exogenous FA intake may be enough to sustain T_reg_ cells (94), future studies can explore if such interplay between FAO and *de novo* lipid synthesis and/or cholesterol catabolism contributes to T_reg_ cell functions. Furthermore, identifying and characterizing the physiological function of additional sterol-related metabolites in T cell responses may provide a novel approach to treat inflammatory diseases.

## Oxygen Sensing

**Figure 4:**
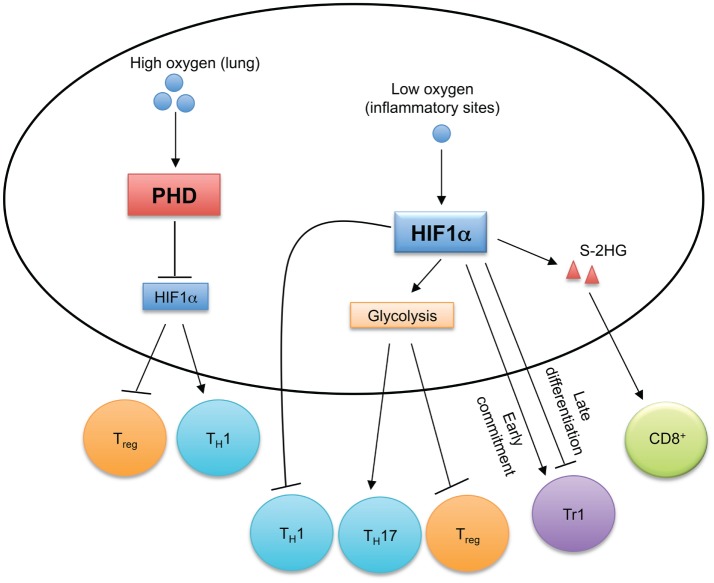
**Oxygen sensing orchestrates T cell immune responses**. In oxygen-replete environments, such as in the lung, prolyl-hydroxylase domain (PHD) proteins sense high levels of cellular oxygen. Activated PHD proteins establish immune tolerance in the lung by inducing hypoxia-inducible factor 1α (HIF1α) degradation to promote T_reg_ cell and inhibit T_H_1 cell differentiation. Hypoxia induces HIF1α expression, which regulates the reciprocal generation of T_H_17 and T_reg_ cells and antagonizes T_H_1 cell differentiation. HIF1α also temporally regulates Tr1 cell differentiation. HIF1α-dependent upregulation of S-2-hydroxyglutarate (S-2HG) enhances anti-tumor CD8^+^ T cell responses.

Oxygen is required for aerobic respiration and mitochondrial energy generation, and the ability for cells to sense and respond to changing oxygen levels promotes their homeostasis. The cellular mechanisms and signaling cascades of oxygen sensing are reviewed elsewhere (95, 96). Reactive oxygen species (ROS), such as peroxides, superoxide, hydroxyl radicals, and singlet oxygen, are byproducts of aerobic metabolism, and the accumulation of ROS causes cellular oxidative stress. ROS plays an important role in regulating T cell responses (97), which will not be discussed. Here, we focus on recent findings on the functions of the oxygen sensing components in T cells (Figure 4).

Prolyl-hydroxylase domain (PHD) proteins are expressed in T cells and function as intracellular oxygen sensors (98). In the lungs, high oxygen levels activate PHD proteins in T cells to suppress T_H_1 responses and promote T_reg_ cell induction (98). However, PHD protein-mediated pulmonary tolerance licenses tumor metastasis to the lungs, and inactivation of PHD proteins promotes anti-tumor T cell responses (98). Mechanistically, activated PHD proteins induce the degradation of HIF1α through post-translational hydroxylation (99), which changes cellular metabolism to promote T_reg_ cell generation and inhibit T_H_1 cell differentiation (98). Sensing of decreased oxygen levels (termed hypoxia) activates HIF1α, which induces glycolysis and provides T cells with the metabolic plasticity to adapt to environmental stress. T cells are recruited to inflammatory sites that are often hypoxic (100), so how HIF1α controls T cell responses has been investigated. HIF1α is dispensable for T cell activation and early metabolic reprogramming *in vitro* (42), and HIF1α-deficient T cells also develop normally *in vivo* (101, 102). However, HIF1α-dependent glycolysis is crucial for promoting T_H_17 cell differentiation and inhibiting T_reg_ cell generation (102). HIF1α also directly interacts with RORγt and Foxp3 to regulate the reciprocal differentiation of T_H_17 and T_reg_ cells (101). HIF1α not only suppresses T_reg_ cell generation (101, 102), but augmented HIF1α activity also impairs T_reg_ cell lineage stability and suppressive function in a VHL or Deltex1-deficient background (103, 104). Although increased HIF1α activity is associated with enhanced T_H_1 cell differentiation in PHD-deficient T cells (98), forced expression of a non-degradable mutant of HIF1α in T_H_1 cells inhibits IFN-γ expression, and T_H_1 cell differentiation is enhanced in HIF1α-deficient T cells after *in vivo* priming (105). Interestingly, HIF1α has differential effects on different stages of type 1 regulatory (Tr1) cell differentiation. Tr1 cells are Foxp3 negative cells that produce IL-10 to control inflammation (106). While HIF1α is required for the metabolic reprogramming during the early commitment of Tr1 cells, its expression reduces at later time points to boost Tr1 cell differentiation by stabilizing AHR expression (107). Augmented HIF1α enhances the effector function of CD8 T cells (108). In the hypoxic tumor microenvironment, the VHL-HIF1α axis regulates CD8^+^ T cell responses by controlling the accumulation of S-2-hydroxyglutarate (S-2HG) in response to TCR stimulation (109). HIF1α promotes the expression of lactate dehydrogenase A that is the enzymatic source of S-2HG (109). S-2HG regulates the differentiation, *in vivo* proliferation and persistence of CD8^+^ T cells, and enhances their anti-tumor efficacy (109). Therefore, by sensing oxygen levels, HIF1α orchestrates T cell responses in given contexts.

## Regulation of Energy and Redox Homeostasis

Energy homeostasis is regulated by dynamic changes in cellular catabolic and anabolic pathways, which generate or consume ATP, respectively. Under conditions of energy and nutrient stress, T cells depend on sensors, such as AMPK, and catabolic pathways, such as autophagy, to replenish energy and cellular resources. Moreover, the redox balance mediated by NAD^+^/NADH is also implicated in T cell responses.

### AMP-Activated Protein Kinase

**Figure 5:**
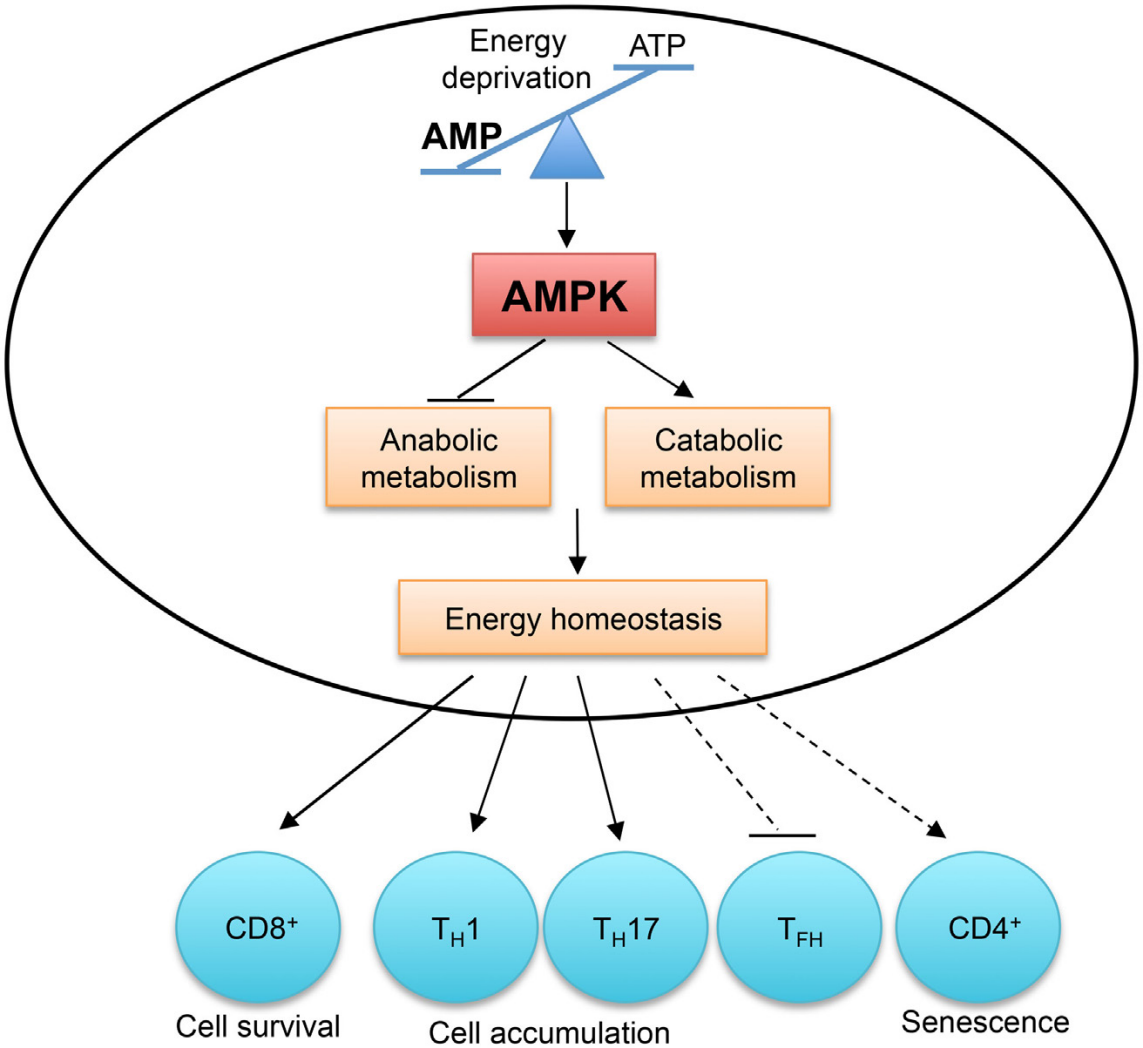
**AMP-activated protein kinase (AMPK) and energy sensing dictate T cell immunity**. AMPK is activated by the increased AMP to ATP ratio, which indicates energy deprivation. AMPK functions as a master regulator of cellular metabolism to preserve cellular energy homeostasis. AMPK activity is required for CD8^+^ T cell survival during infection and in tumor microenvironment. The accumulation of T_H_1 and T_H_17 cells in the colon is also dependent on AMPK activity. Aberrant AMPK activation is linked to impaired T_FH_ cell generation and CD4^+^ T cell senescence.

Energy homeostasis is the basis of cell survival and function. AMPK is directly activated by an increased cellular AMP to ATP concentration during energy deprivation. AMPK functions as a fundamental regulator of T cell metabolism to inhibit anabolic pathways and to promote catabolic pathways to preserve cellular energy homeostasis (110). While the activation, regulation, and signaling of AMPK has been reviewed elsewhere (7, 111), here we will briefly discuss the functions of AMPK in T cells (Figure 5). AMPK is activated in glucose-deprived CD8^+^ T cells experiencing energy stress, such as during infection and inflammation. AMPK is critical for both primary and memory CD8^+^ T cell responses to infections (112, 113). In nutrient-limiting tumor microenvironment, CD8^+^ T cells also require AMPK activation for their survival to defend against tumors (114). The pro-survival functions of AMPK could also be linked to the induction of macroautophagy, which is discussed in detail below (115).

In activated T cells, AMPK is a master regulator of cellular metabolism. It preserves energy homeostasis by suppressing mRNA translation and promoting glutamine-fueled mitochondrial oxidization in response to glucose deprivation (112). The differentiation of T_H_1 and T_H_17 cells from naïve CD4^+^ T cells does not require AMPK activity, but their accumulation and their ability to induce inflammation in the colon are severely impaired in the absence of AMPK (112). However, AMPK activation is detrimental to T cell responses in certain contexts. In response to nutrient stress and DNA damage, active AMPK induces p38-dependent senescence in human CD4^+^ T cells, and silencing of AMPK restores telomerase activity and proliferation in senescent T cells (116). Aberrant AMPK activation is linked to impaired T_FH_ cell generation in T cells deficient in the ROQUIN RING domain (117). The AMPK agonist, 5-aminoimidazole-4-carboxamide ribonucleotide (AICAR), inhibits T_H_17 cell induction and promotes T_reg_ cell generation *in vitro* (118), but these results should be interpreted cautiously due to the reported off-target effects of AICAR (119). Therefore, by sensing energy stress, AMPK plays a pivotal role in metabolic reprogramming to regulate T cell responses.

## NAD^+^/NADH and Redox Balance

The coenzyme β-nicotinamide adenine dinucleotide (NAD^+^) and its reduced form, NADH, are critical regulators of T cell biology. Early studies demonstrated that NAD^+^ induces apoptosis in T cells in a process called NAD^+^-induced cell death (NICD). When cells die as a result of tissue damage or inflammation, intracellular NAD^+^ is released into microenvironment, which then activates signaling to induce cell death. In T cells, the accumulation of NAD^+^ increases ADP-ribosylation mediated by mono-ADP-ribosyltransferase 2 (ART2) (120). The purinergic receptor P2X7 is one substrate of ART2, and its ADP-ribosylation increases its sensitivity for activation by extracellular ATP, which initiates the cell death program (120). Consistent with this observation, elevated NAD^+^ levels generated during acute inflammation result in ART2-dependent decrease in peripheral CD4^+^ and CD8^+^ T cell numbers (121). Interestingly, peripheral T cells are more sensitive to NICD than thymocytes (120), suggesting a more dominant role for NAD^+^ in regulating peripheral T cell homeostasis. Compared to conventional T cells, T_reg_ cells express higher levels of ART2 and P2X7 and are more sensitive to NICD (122). *In vivo* NAD^+^ administration preferentially depletes T_reg_ cells and enhances anti-tumor T cell responses (122). Thus, localized changes in NAD^+^ levels likely influence the balance between conventional and T_reg_ cell responses.

The flux of electrons *via* mitochondrial electron transport chain (ETC) regulates NAD^+^/NADH redox balance important for maintaining mitochondrial function and energy metabolism (123). Furthermore, perturbations in ETC alter NAD^+^/NADH levels and attenuate T cell proliferation. Recently, mechanisms linking ETC dysfunction and impaired proliferation of Jurkat leukemic T cells were uncovered. Disruption of ETC alters NAD^+^/NADH redox balance and limits mitochondrial aspartate synthesis, a process linked to purine and pyrimidine biosynthesis and protein synthesis (124). Pyruvate supplementation restores NAD^+^ levels and increases aspartate aminotransferase (GOT1)-dependent aspartate synthesis (124). However, when *GOT1* (encodes for GOT1) is deleted from Jurkat cells, NAD^+^ levels and aspartate levels are further depleted, leading to increased Jurkat cell arrest following ETC inhibition (124). Thus, aspartate synthesis is limiting for proliferation when ETC is inhibited. The regulatory role of aspartate biosynthesis in primary T cell development and function is unexplored; however, because aspartate synthesis is linked to purine nucleotide biosynthesis important for T cell proliferation (56), this pathway likely plays an important role in these cells.

Recent studies highlight the role of NAD^+^ in regulating CD4^+^ T cell differentiation. NAD^+^ regeneration through mitochondrial respiration is essential for proper lysosome function, which restrains excessive T_H_1 cell generation both *in vivo* and *in vitro* (125). NAD^+^ treatment blocks the induction of EAE in mice by promoting the generation of immunosuppressive IFN-γ^+^IL-10^+^ CD4^+^ T cells (126). Collectively, these studies highlight the potential for novel NAD^+^-based immune modulation.

### Autophagy

Macroautophagy, hereafter referred to as autophagy, is an evolutionarily conserved cellular process that breaks down endogenous cytosolic materials and organelles (127). Autophagy helps regulate energy consumption and cellular resources during cellular stress. Indeed, amino acid starvation and other energy-stressing conditions induce autophagy, which in part depends upon AMPK and mTORC1 signaling (128). How nutrient availability regulates autophagy in T cells remains elusive. Earlier *in vitro* studies showed evidence for increased autophagy in T cells after TCR and cytokine stimulation (129–131). However, *in vivo*, virus-specific CD8^+^ T cells downregulate autophagy during clonal expansion (132). In contrast, during the contraction phase, autophagy is upregulated in virus-specific CD8^+^ T cells (132). Furthermore, upregulation of autophagy is critical for CD8^+^ T cell memory formation, as acute deletion of *Atg7* or *Atg5* in peripheral CD8^+^ T cells impairs survival during the contraction phase, resulting in a decreased memory CD8^+^ T cell pool (132). Thus, the regulation of autophagy during the kinetics of T cell responses is dynamic. The contribution of cellular stress conditions, such as nutrient levels, growth factor withdrawal, energy, and oxidative stress, in regulating autophagy and effector T cell responses remains to be further elucidated.

Recent studies have shown that autophagy regulates CD4^+^ T cell lineage differentiation and functionality. T_reg_ cells have increased autophagosomes compared to conventional CD4^+^ T cells (33, 133, 134). T_reg_ cell-specific deletion of genes required for autophagy (*Atg7, Atg5, Atg16l1*) reduces T_reg_ cell survival, stability of Foxp3 expression and ability to control pro-inflammatory CD4^+^ T cell responses (33, 133). Furthermore, loss of autophagy in T_reg_ cells aberrantly elevates mTORC1 and c-Myc expression, which enhances glycolysis and limits T_reg_ cell stability and function (33). This is in contrast to autophagy-dependent reactivation of mTORC1 signaling under conditions of sustained nutrient deprivation (135). Interestingly, the microenvironment may alter the role of autophagy in T cells, as *Atg16l1*-deficient CD4^+^ T cells had elevated T_H_2 cell responses selectively within the intestinal environment (133). How autophagy regulates mTORC1 signaling is not completely understood. In T_reg_ cells, autophagy may selectively target PI(3)K-related signaling proteins for degradation (33). In effector T cells, autophagy selectively degrades Bcl-10, affecting the signaling between TCR and NF-κB (136). Interestingly, the CARMA1/Bcl-10/MALT1 complex contributes to the upregulation of ASCT2 expression and glutamine uptake in TCR activated T cells, which controls mTORC1 activation (41). Thus, the role of autophagy in T cells is complex, as it not only regulates cellular degradation in times of nutrient deprivation but also serves to modulate TCR signaling.

## Concluding Remarks

While it is clear that the interplay of nutrient sensing, signaling proteins, and cellular metabolism controls T cell functional fitness, it remains an open question as to how T cells sense dynamic nutrient cues in complex inflammatory contexts. Recent studies have begun to identify specific nutrients, nutrient sensors, and nutrient transporters that are crucial for proper T cell immune responses. However, the importance of additional sensors identified in other cellular systems remains to be explored in T cells. For example, SLC38A9 and Castor1 are proteins associated with the lysosomes that directly bind intracellular arginine, leading to downstream regulation of mTORC1 signaling (52–54). Recent studies have also shown that ion sensing regulates T cell immunity (137, 138), but how ion and other nutrients cooperate to control T cell responses remains to be established. One major challenge in studying nutrient and energy sensing is to identify key molecules involved in these processes. The development of cutting-edge, gene-targeting technologies, such as CRISPR, will likely allow us to identify additional molecules that specifically control selective aspects of T cell functions. Furthermore, the complex interplay between nutrient and cellular stress signals should also be explored in the context of cancer, autoimmunity, and infectious diseases to gain a better understanding of how T cell functional responses adapt to different physiological and pathological conditions. A detailed understanding of nutrient sensing in T cells has the potential to be translated into innovative therapies for immune-mediated diseases and cancer.

## Author Contributions

JW wrote the manuscript and organized the review. JR and T-LN wrote part of the manuscript. HC edited the manuscript and provided direction.

## Conflict of Interest Statement

The authors declare that the research was conducted in the absence of any commercial or financial relationships that could be construed as a potential conflict of interest.
